# Chikungunya Virus Infection and Acute Elevation of Serum Prostate-Specific Antigen

**DOI:** 10.1155/2015/120535

**Published:** 2015-06-17

**Authors:** William Derval Aiken, Joshua J. Anzinger

**Affiliations:** ^1^Division of Urology, Section of Surgery, Department of Surgery, Anaesthesia & Intensive Care and Emergency Medicine, Faculty of Medical Sciences, University of the West Indies, Mona, Kingston 7, Jamaica; ^2^Department of Microbiology, Faculty of Medical Sciences, University of the West Indies, Mona, Kingston 7, Jamaica

## Abstract

A man with prostate cancer on a regime of active surveillance had a laboratory-confirmed acute Chikungunya virus infection. The patient experienced a sudden increase in serum Prostate-Specific Antigen (PSA) during the acute illness that caused him anxiety and confounded interpretation of the PSA test. Six weeks after the onset of Chikungunya Fever symptoms, the elevated serum PSA returned to baseline. The association of Chikungunya Fever and elevated serum PSA may result in misinterpretation of the PSA test, triggering unnecessary prostate biopsy or other management errors.

## 1. Introduction

The Caribbean recently experienced an epidemic of Chikungunya Fever, a mosquito-borne infection caused by the Chikungunya virus that is [1] commonly associated with abrupt onset of fever, asthenia, headache, and debilitating joint pain [2, 3] after a 2–10 day incubation period. Approximately 50% of infected persons also experience a generalized pruritic rash [4]. Acute symptoms occur for 1–4 days and are associated with viremia that usually resolves 6 days after the onset of symptoms [5]. Chronic joint pain lasting greater than a month is commonly observed in most [6–9] but not all [10] populations studied. In one study, most chronic infections were found to occur in persons ≥30 years of age [7]. Chronic joint pain can persist after the acute viral infection or can reoccur after the resolution of acute symptoms.

Chikungunya virus continues to disseminate beyond the Caribbean to other regions within the Americas [3], dramatically extending affected areas beyond the Eastern Hemisphere. Most countries in the world have now reported Chikungunya virus infections (http://www.cdc.gov/chikungunya/geo/index.html), and genetic mutation [11–15] and climate change [16] could further increase geographical dissemination of the virus to unaffected regions.

During the recent Chikungunya Fever epidemic in the Caribbean, a patient residing in Kingston, Jamaica, was being monitored for prostate adenocarcinoma. He presented with clinical symptoms of Chikungunya Fever and a concomitant, dramatic elevation of serum Prostate-Specific Antigen (PSA), a commonly used biomarker to screen patients for prostate cancer. Here, we report on a possible association between acute Chikungunya virus infection and elevation of serum PSA.

## 2. Case Presentation

**Figure 1:**
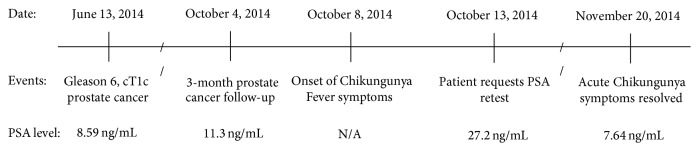
Timeline of PSA changes in relation to Chikungunya infection.

A 64-year-old Afro-Caribbean man diagnosed with Gleason 6 (3 + 3) adenocarcinoma of the prostate involving 5% of 1 of 12 cores arising from the left lobe of the prostate and with a prebiopsy PSA level of 8.59 ng/mL (June 13, 2014) and cT1c (clinically benign) prostate on digital rectal examination (DRE) went on a regime of active surveillance after diagnosis on July 14, 2014. The patient was advised to return 3 months later for a follow-up appointment (see Figure 1 for timeline of events).

### 2.1. Three Months Later

On October 4, 2014, the patient's PSA level was 11.3 ng/mL. Four days later (October 8, 2014), he developed acute symptoms of fever, rash on the trunk and proximal limbs, headaches, and then severe joint pain. After notification of the PSA test results, the patient was alarmed by the rise in PSA compared to the level at the time of diagnosis of prostate adenocarcinoma and requested that the PSA test be repeated by another laboratory, which reported a PSA level of 27.2 ng/mL on October 13, 2014.

When seen, he denied having any lower urinary tract symptoms (LUTS) apart from increased urinary frequency which he attributed to increased fluid intake. DRE was unchanged and the prostate was nontender. Given the sudden rise in the PSA, he was diagnosed as having subclinical prostatitis possibly related to Chikungunya infection. The patient was advised to test for Chikungunya and Dengue virus infection and repeat PSA testing in 4 weeks.

### 2.2. Four Weeks Later

The patient was seen 5 weeks later on November 26, 2014, and he complained of ongoing joint pain and swelling of the hands and ankles which was so severe; it necessitated a steroid injection 2 days earlier. He had no LUTS and repeat PSA was 7.64 ng/mL on November 20, 2014. Recent Chikungunya virus infection was confirmed from a serum sample taken on December 1, 2014, by the presence of Chikungunya virus-specific IgM. Dengue virus-specific IgM was not present, indicating that recent infection with Dengue virus was unlikely.

## 3. Discussion

This is the first report of a possible association between Chikungunya virus infection and a sudden rise in PSA that could be due to subclinical prostatitis. Although the pathogenic mechanism responsible for the increase in PSA during Chikungunya virus infection is unknown, microbes implicated in prostatitis are believed to cause an increase in serum PSA through one of three mechanisms that are not mutually exclusive. These include leakage of PSA into the bloodstream, increased blood flow through the prostate, and increased vascular permeability [17].

If an association between acute Chikungunya virus infection and sudden elevations of the PSA is present, it would be important for clinicians to be aware of it, as it may confound the interpretation of PSA, causing unnecessary patient anxiety, and lead to unnecessary and invasive tests, or worse a change in patient management.
